# Point of Care Ultrasound in Pyogenic Tenosynovitis: A Case Report

**DOI:** 10.29252/beat-080107

**Published:** 2020-01

**Authors:** Richard Amini, Luis Camacho, Josie Acuña, Elaine H Situ-La Casse, Srikar Adhikari

**Affiliations:** 1 *Department of Emergency Medicine, The University of Arizona, Tucson, AZ, USA*; 2 *College of Medicine, The University of Arizona, Tucson, AZ, USA*

**Keywords:** Tenosynovitis, Pyogenic Tenosynovitis, Septic Tenosynovitis, Point of Care, Bedside Ultrasound

## Abstract

Pyogenic tenosynovitis is caused by hematogenous spread of infection or trauma with direct inoculation of a tendon sheath. Symptoms and clinical examination findings associated with pyogenic tenosynovitis may be confused with superficial soft tissue infections, however management plans between pyogenic tenosynovitis and superficial soft tissue infection vary significantly. In patients with pyogenic tenosynovitis, operative intervention and subsequent irrigation and debridement offer a definitive therapy. Bedside ultrasound helps clinicians inspect the involved tendon sheath and may help assisting diagnosis of pyogenic tenosynovitis. In this case report, we described three cases, where point of care ultrasound was used to assist the diagnosis of pyogenic tenosynovitis, to accelerate consultation, and to expedite operative intervention.

## Introduction

Tenosynovitis is defined as inflammation of a tendon and its synovial sheath. Pyogenic tenosynovitis is often caused by hematogenous spread of infection or by trauma with direct inoculation of a tendon. A high-pressure environment may develop, due to increased purulence contained within the tendon sheath, which impedes vascular flow to the tendon leading to ischemia and necrosis [1, 2]. Symptoms and clinical examination findings associated with pyogenic tenosynovitis may be confused with superficial soft tissue infections as both include pain over the affected digit or tendon sheath region, tenderness with movement of associated musculoskeletal anatomy, and soft tissue fullness or swelling [3]. 

These symptoms commonly are present in superficial infections such as cellulitis, felons, and traumatic injuries; however, management plans vary significantly. In patients with pyogenic tenosynovitis, operative intervention provides surgeons visual confirmation of the disease process and the subsequent irrigation and debridement offers definitive therapy [4]. Point of care ultrasound (US) has become ubiquitous within emergency departments (ED), and most ED’s have a dedicated ultrasound machine available at all times. Ultrasound imaging for soft tissue infections was shown to improve diagnostic accuracy and alter management plans [5, 6] and can be utilized for tendon injury and fluid collection assessment [7]. 

In the case of pyogenic tenosynovitis, the application of bedside ultrasound can provide clinicians the opportunity to visually inspect the involved tendon sheath and can assist with the management, when the diagnosis is unclear. In this case report, we described three cases where point of care ultrasound was used in the ED to assist the diagnosis of pyogenic tenosynovitis, accelerate consultation, and expedite operative intervention.

**CASE 1**

The patient was a 29-year-old female with a past medical history of anxiety, insomnia, and intravenous drug abuse who presented to the ED with left ankle and foot swelling and pain for five days. The patient reported that she injured her left foot while rock-climbing five days prior to presenting to the ED. She started having pain and swelling two days after the rock-climbing injury and was subsequently seen at a local urgent care, where radiographs of the foot were performed and resulted negative for acute fracture. 

The patient was discharged with crutches and a compression wrap. Her symptoms subsequently worsened and she began to develop fevers at home. Vital signs on presentation were blood pressure (BP) of 124/66 mmHg, heart rate (HR) of 90 beats/min, respiratory rate (RR) of 18 breaths/min, oxygen saturation of 100% on room air (RA), and temperature of 36.9°C. Physical examination revealed an erythematous left ankle with swelling along the medial aspect of the ankle. 

**Fig. 1:**
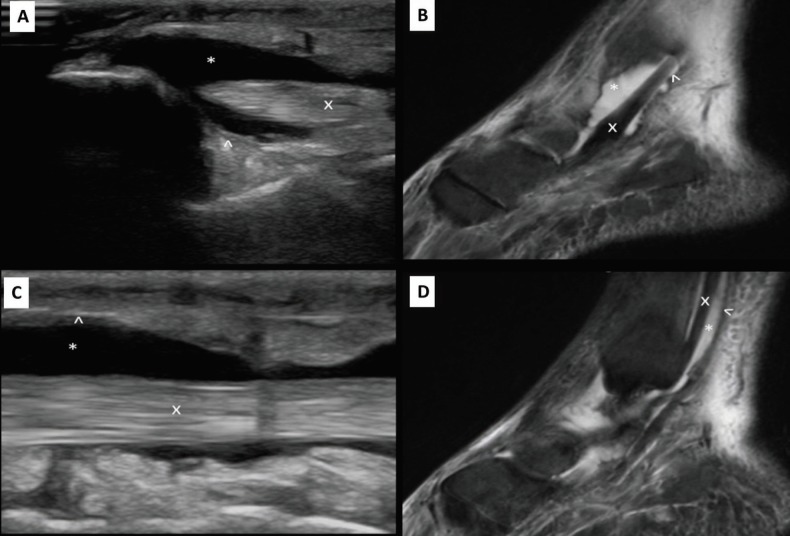
**A.** Sagittal ultrasound image of the affected left ankle flexor tendons near the medial malleolus demonstrating synovial fluid collection above and below the tendon was demonstrated. **B.** Sagittal magnetic resonance image of the affected left ankle flexor tendons near the medial malleolus demonstrating synovial fluid collection above and below the tendon was illustrated. **C.** Sagittal ultrasound image of the affected flexor tendon sheath demonstrating circumferential synovial fluid collection was displayed. **D.** Sagittal magnetic resonance image of the affected flexor tendon sheath demonstrating circumferential synovial fluid collection was shown. **Index: **“x” identifies the tendon. “^’ identifies the tendon sheath. “*” Identifies the tendon sheath fluid

Radiographs of the left ankle and foot were positive for soft tissue swelling, but negative for joint effusion or fracture. Relevant laboratory findings were as follows: WBC: 15.1 1,000/uL, CRP: 29.7 mg/dL, ESR: 76 MM/HR, Hemoglobin: 12.3 g/dL, and Glucose: 96 mg/dL. Point of care ultrasound performed by the emergency physicians demonstrated minimal soft tissue edema; however, there was significant amounts of fluid around the synovium of the flexor tendons at the medial ankle, which was concerning for tendon sheath infection (Figure 1A and 1C). 

The orthopedic surgery service was consulted due to concern for pyogenic tenosynovitis and the patient was admitted and started on broad-spectrum antibiotics. A magnetic resonance imaging (MRI) study was subsequently ordered by orthopedic surgeons preoperatively, which demonstrated tenosynovitis of the left ankle flexor tendons, most pronounced in the posterior tibialis tendon sheath (Figure 1B and 1D). The patient was taken to the operating room (OR) for debridement and recovered well with an uncomplicated post-operative hospital course. We had IRB approval for this case report.

**CASE 2**

The patient was a 42-year-old female with a significant past medical history for diabetes who presented to the ED for a wound check. A week prior, the patient had been bitten on her right thumb by a cat. She presented to an urgent care at this time and was started on Augmentin. On the day of her visit, patient described waking up to an increase in swelling and redness to the right hand. Vital signs on presentation were BP of 135/82 mm Hg, HR of 82 beats/minute, RR of 16 breaths/minute, oxygen saturation of 98% on RA, and Temperature of 36.5°C. 

**Fig. 2:**
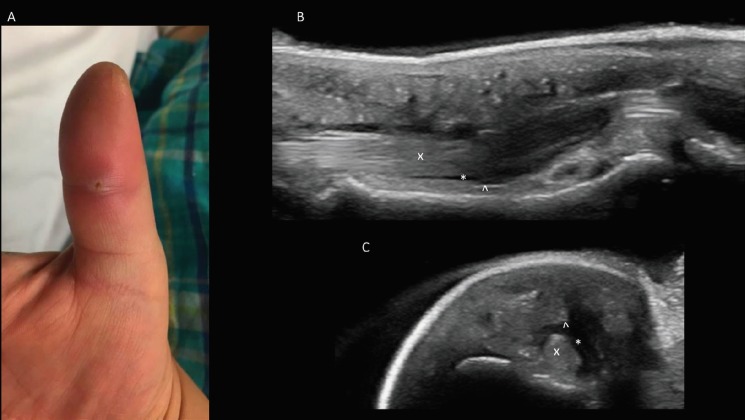
**A.** Photograph of affected Right Thumb was shown. **B.** Sagittal volar ultrasound image of the affected digit demonstrating synovial fluid collection above and below the tendon was demonstrated. **C.** Transverse volar ultrasound image of the affected digit demonstrating circumferential synovial fluid collection was illustrated. **Index: **“x” identifies the tendon. “^’ identifies the tendon sheath. “*” Identifies the tendon sheath fluid

**Fig. 3:**
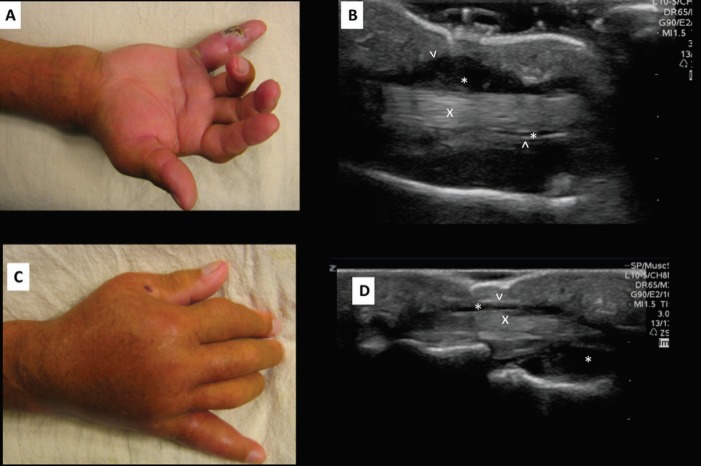
**A.** Volar photograph of affected right hand was shown. B. Sagittal volar ultrasound image of the affected digit demonstrating synovial fluid collection above and below the tendon was displayed. **C.** Dorsal photograph of affected right hand was demonstrated. **D.** Sagittal dorsal ultrasound image of the affected digit demonstrating synovial fluid collection above and below the tendon was illustrated. **Index: **“x” identifies the tendon. “^’ identifies the tendon sheath. “*” Identifies the tendon sheath fluid

**Table 1:** Bedside ultrasound compared to other imaging modalities for various pathologies

**Study**	**Pathology**	**Ultrasound**		**CT**		**MRI**	
		**Sensitivity** **(%)**	**Specificity** **(%)**	**Sensitivity** **(%)**	**Specificity** **(%)**	**Sensitivity** **(%)**	**Specificity** **(%)**
Gaspari et al.	Skin abscess	96.7	85.7	76.7	91.4	-	-
Mohty et al.	Necrotizing fasciitis	88	93	-	-	43	100
Martín-Hervás et al.	Tendinitis	58	100	-	-	81	97

The patient exhibited marked tenderness to palpation over the volar aspect of the right thumb. A puncture wound was noted over the distal interphalangeal joint of the dorsal thumb. There was normal sensation and strength testing throughout the right hand. An x-ray of the right hand was performed, which demonstrated no acute fracture or dislocation. There was only mild soft tissue edema noted around the thumb (Figure 2A). Relevant laboratory findings were as follows: WBC: 11.0 1,000/uL, Hemoglobin: 15.8 g/dL, Glucose: 197 mg/dL. Point of care ultrasound performed by the emergency physicians demonstrated fluid surrounding the extensor tendon (Figures 2B and 2C).

Although the surrounding tissue demonstrated sonographic cobble-stoning consistent with cellulitis, the circumferential fluid noted surrounding the tendon sheath increased the clinician’s concern for pyogenic tenosynovitis. As a result, hand surgery was consulted for further evaluation. Patient was then taken to the OR for irrigation and debridement of the right thumb and recovered well with an uncomplicated post-operative hospital course. We had IRB approval for this case report.

**CASE 3**

The patient was a 56-year-old male with a past medical history, significant for poorly controlled diabetes who presented to the ED with a complaint of right sided hand pain and swelling for the past two days. The patient described having a non-healing abrasion to the right fifth digit. He recently began noticing a sudden increase of swelling and pain throughout the entire hand. Vital signs on presentation were BP of 132/69 mm Hg, HR of 93 beats/minute, RR of 18 breaths/minute, oxygen saturation of 98% on RA, and Temperature of 37.9°C. 

On physical examination, the patient lacked any immediate distress. Examination of the right hand revealed significant swelling over the dorsal aspect of the right hand and wrist with an edematous and erythematous fifth digit (Figures 3A and 3C). There was an associated abrasion to the lateral aspect of the fifth digit with purulent drainage produced upon palpation. The fourth digit was notable for fusiform swelling and was found to be in a flexed position. The patient exhibited tenderness to palpation over the flexor tendons as well as discomfort with manipulation of the digits. 

Relevant laboratory findings were as follows: WBC: 13.0 1,000/uL, Hemoglobin: 13.5 g/dL, Glucose: 210 mg/dL. Radiographs of the right hand demonstrated extensive soft tissue edema, most prominently about the dorsum of the hand and metacarpophalangeal joints. ED point of care ultrasound demonstrated mild soft tissue edema; however, significant fluid surrounding the flexor tendon of the fourth digit was noted (Figures 3B and 3D), and thus increased concern for pyogenic tenosynovitis. Orthopedic surgery was consulted and the patient was taken to OR for surgical irrigation and debridement of fourth and fifth digits. We had IRB approval for this case report.

## Discussion

Pyogenic tenosynovitis is often caused by hematogenous spread of infection or trauma with direct inoculation. Pyogenic tenosynovitis develops when purulent fluid accumulates between the visceral and parietal synovial layers of the tendon. In the setting of increased purulence, a high-pressure environment can impede vascular flow to the tendon itself leading to ischemia and necrosis [1, 2]. Risk factors associated with poor outcomes include an age of over 40 years, diabetes mellitus, renal failure, peripheral vascular disease, local ischemia, presence of subcutaneous purulence, and polymicrobial infections. 

Furthermore, patients with extensive subcutaneous purulence and ischemic changes had an amputation rate of 59% [3]. Surgical intervention, i.e. debridement and irrigation, with prolonged antibiotic therapy is the definitive management in these cases. Early diagnosis and management can mitigate complications such as decreased range of motion, recurrent infections, or amputations [4]. Symptoms and clinical examination findings associated with pyogenic tenosynovitis can be confused with superficial soft tissue infections as both include pain over the affected digit or tendon sheath region, tenderness with movement of associated musculoskeletal anatomy, and soft tissue fullness or swelling [3]. 

The resulting differential diagnosis includes soft tissue abscesses, felons, gouty arthritis, pyogenic arthritis, and even herpetic whitlow. Although infections involving the flexor tendons of the hands can present with “Kanavel signs:” affected finger held in slight flexion, uniform swelling of the digit, pain with extension of digit, and tenderness over the affected tendon; a recent meta-analysis demonstrated that these cardinal signs have a specificity of only 51-69% [8]. Furthermore, a 2015 retrospective study of 126 cases demonstrated that the only predictive finding associated with patients requiring operative intervention in pyogenic tenosynovitis was the presence of an abscess during the initial evaluation [4]. 

Infection of tendons in the lower extremities is less common and even more difficult to diagnose by physical examination alone. Imaging modalities such as magnetic resonance imaging (MRI) and computed tomography (CT) have often been required to arrive at a diagnosis [1]. Unfortunately, these imaging studies may lengthen the time to diagnosis and the time to definitive management, resulting in increased risk of morbidity. Due to the diagnostic challenge and risk for significant complications, emergency clinicians often rely on consultation with orthopedics and, at times, transfer to another facility with orthopedic coverage.

In the diagnosis of pyogenic tenosynovitis, ultrasound has proven to be more sensitive than the clinical examination alone for detecting tenosynovitis [9]. Ultrasound findings consistent with the diagnosis of pyogenic tenosynovitis include tendon thickening of greater than 25%, fluid accumulation within the tendon sheath, and echogenic material within the synovial fluid [10]. These findings require a relatively basic understanding of sonographic anatomy of tendons and tendon sheaths [11]. 

Tendons are seen as multiple echogenic parallel lines on longitudinal scanning and multiple echogenic fibers on sagittal plane imaging (Figure 1A and 1C). As mentioned above, an inflamed tendon will become edematous and >25% increase in transverse diameter measurement is consistent with inflammation. Furthermore, sonographic evaluation demonstrates the loss of normal fibrillar structure resulting from increased spacing of the hyperechoic fibrillar lines due to thickening of the tendon caused by inflammation. 

The tendon sheath is rarely visible, however when bacteria grow in this space, purulent bi-products begin to separate the visceral and parietal synovial layers and an anechoic (black) fluid collection may be visible (Figure 1C) [11]. Ultrasound has various advantages over other more time-consuming imaging modalities in this setting. A retrospective review of 65 patients receiving both soft tissue bedside ultrasound and a CT for suspected skin abscesses demonstrated how ultrasound is significantly more sensitive than CT (96.7% vs. 76.7%), but only slightly less specific than CT (85.7% vs. 91.4%) [5]. 

Additionally, a recent review demonstrated that ultrasound is more sensitive than MRI (88% vs. 43%), but less specific (93% vs. 100%) for diagnosing necrotizing fasciitis [12]. Lastly, another study demonstrated that ultrasound is more specific (100% vs. 97%), but less sensitive (58% vs. 81%) than MRI for detecting tendinitis [13]. Point of care ultrasound has been successfully integrated into the curriculum of nearly all emergency medicine residencies and is available in nearly every ED in the country (Table 1) [13, 14]. 

Point of care ultrasound is fast, radiation free, and, due to its increased sensitivity, should be the imaging modality of choice in patients with suspected pyogenic tenosynovitis. The diagnostic sensitivity and specificity of ultrasound has been demonstrated to be similar to CT and MRI, however due to its availability and speed, it can expedite patient care and minimize time to operative management. Furthermore, in health care settings with limited access to CT and MRI imaging, or where patient transfer is contingent on diagnostic confidence, ultrasound has the potential of significantly improving the morbidity and mortality associated with pyogenic tenosynovitis. 

In all three cases demonstrated above, the emergency clinician was able to perform a bedside ultrasound and detect sonographic findings suggestive of pyogenic tenosynovitis. In all three cases, consultation with orthopedics was expedited as a result of the bedside ultrasound. Finally, all three cases resulted in positive clinical outcomes and uncomplicated post-operative courses too.

## Conclusion

Our case report illustrated how point of care ultrasound is used in the ED for the diagnosis and prompt treatment of pyogenic tenosynovitis. Clinicians should be aware of the unique presentations of pyogenic tenosynovitis as well as the utility of ultrasound in the diagnosis of such pathology.

## Funding/Support:

None

## Other disclosures:

None

## Disclosures:

The authors have nothing relevant to disclose.

## Conflict of interests:

The authors have no conflicts of interests to report.

## References

[1] Schecter WP, Markison RE, Jeffrey RB, Barton RM, Laing F (1989). Use of sonography in the early detection of suppurative flexor tenosynovitis. J Hand Surg Am..

[2] Schnall SB, Vu-Rose T, Holtom PD, Doyle B, Stevanovic M (1996). Tissue pressures in pyogenic flexor tenosynovitis of the finger Compartment syndrome and its management. J Bone Joint Surg Br..

[3] Pang HN, Teoh LC, Yam AK, Lee JY, Puhaindran ME, Tan AB (2007). Factors affecting the prognosis of pyogenic flexor tenosynovitis. J Bone Joint Surg Am..

[4] Müller CT, Uçkay I, Erba P, Lipsky BA, Hoffmeyer P, Beaulieu JY (2015). Septic Tenosynovitis of the Hand: Factors Predicting Need for Subsequent Débridement. Plast Reconstr Surg..

[5] Gaspari R, Dayno M, Briones J, Blehar D (2012). Comparison of computerized tomography and ultrasound for diagnosing soft tissue abscesses. Crit Ultrasound J..

[6] Tayal VS, Hasan N, Norton HJ, Tomaszewski CA (2006). The effect of soft-tissue ultrasound on the management of cellulitis in the emergency department. Acad Emerg Med..

[7] Situ-LaCasse E, Grieger RW, Crabbe S, Waterbrook AL, Friedman L, Adhikari S (2018). Utility of point-of-care musculoskeletal ultrasound in the evaluation of emergency department musculoskeletal pathology. World J Emerg Med..

[8] Kennedy CD, Lauder AS, Pribaz JR, Kennedy SA (2017). Differentiation Between Pyogenic Flexor Tenosynovitis and Other Finger Infections. Hand (N Y)..

[9] Hmamouchi I, Bahiri R, Srifi N, Aktaou S, Abouqal R, Hajjaj-Hassouni N (2011). A comparison of ultrasound and clinical examination in the detection of flexor tenosynovitis in early arthritis. BMC Musculoskelet Disord..

[10] Padrez K, Bress J, Johnson B, Nagdev A (2015). Bedside ultrasound identification of infectious flexor tenosynovitis in the emergency department. West J Emerg Med..

[11] Chau CL, Griffith JF (2005). Musculoskeletal infections: ultrasound appearances. Clin Radiol..

[12] Mohty KM, Cravens MG, Adamas-Rappaport WJ, Amini-Shervin B, Irving SC, Stea N, Adhikari S, Amini R (2017). Cadaver-based Necrotizing Fasciitis Model for Medical Training. Cureus..

[13] Martín-Hervás C, Romero J, Navas-Acién A, Reboiras JJ, Munuera L (2001). Ultrasonographic and magnetic resonance images of rotator cuff lesions compared with arthroscopy or open surgery findings. J Shoulder Elbow Surg..

[14] Moore CL, Molina AA, Lin H (2006). Ultrasonography in community emergency departments in the United States: access to ultrasonography performed by consultants and status of emergency physician-performed ultrasonography. Ann Emerg Med..

